# 8-Gene signature related to CD8^+^ T cell infiltration by integrating single-cell and bulk RNA-sequencing in head and neck squamous cell carcinoma

**DOI:** 10.3389/fgene.2022.938611

**Published:** 2022-07-22

**Authors:** Shoujing Zhang, Wenyi Zhang, Jian Zhang

**Affiliations:** ^**1**^ Department of Oral and Maxillofacial Surgery, Tianjin Medical University School and Hospital of Stomatology, Tianjin, China; ^**2**^ Department of Prosthodontics, Tianjin Medical University School and Hospital of Stomatology, Tianjin, China

**Keywords:** CD8^+^ T cells, head and neck squamous cell carcinoma, immunotherapy, prognosis, weighted gene co-expression network analysis

## Abstract

**Background:** CD8^+^ T cells, a critical component of the tumor immune microenvironment, have become a key target of cancer immunotherapy. Considering the deficiency of robust biomarkers for head and neck squamous cell carcinoma (HNSCC), this study aimed at establishing a molecular signature associated with CD8+T cells infiltration.

**Methods:** Single-cell RNA sequencing data retrieved from the Gene Expression Omnibus (GEO) database was analyzed to obtain the different cell types. Next, the cell proportions were investigated through deconvolution of RNA sequencing in the Cancer Genome Atlas (TCGA) database, and then the immune-related genes (IRGs) were identified by weighted gene co-expression network analysis (WGCNA). LASSO-Cox analysis was employed to establish a gene signature, followed by validation using a GEO dataset. Finally, the molecular and immunological properties, and drug responses between two subgroups were explored by applying “CIBERSORT”, “ESTIMATE”, and single sample gene set enrichment analysis (ssGSEA) methods.

**Results:** A total of 215 differentially expressed IRGs were identified, of which 45 were associated with the overall survival of HNSCC. A risk model was then established based on eight genes, including *DEFB1*, *AICDA*, *TYK2*, *CCR7*, *SCARB1*, *ULBP2*, *STC2*, and *LGR5*. The low-risk group presented higher infiltration of memory activated CD4^+^ T cells, CD8^+^ T cells, and plasma cells, as well as a higher immune score, suggesting that they could benefit more from immunotherapy. On the other hand, the high-risk group showed higher abundance of activated mast cells and M2 macrophages, as well as a lower immune score.

**Conclusion:** It was evident that the 8-gene signature could accurately predict HNSCC prognosis and thus it may serve as an index for clinical treatment.

## Introduction

Head and neck squamous cell carcinoma (HNSCC) is the seventh most common malignancy worldwide (Siegel et al., 2020). Despite the effective and aggressive treatment strategies involving surgery combined with radio- and chemotherapy, patients with advanced stage HNSCC only have a 50% five-year survival rate (Vigneswaran and Williams, 2014). In recent years, immunotherapy involving checkpoint inhibitors blocking programmed cell death protein 1 (*PD-1*) or programmed death ligand-1 (*PD-L1*) has been approved for clinical use, with preliminary results showing that the strategy significantly improves the overall survival of recurrent or metastatic HNSCC patients. However, several clinical trials have demonstrated that anti-*PD-1*/*PD-L1* therapy is only beneficial to a few patients (Ferris et al., 2016; Siu et al., 2019). Studies have suggested that CD8^+^ T lymphocytes substantially express *PD-1* and may play an important role in the efficacy of immunotherapy (Jia et al., 2020). It is worth noting that high dense infiltration of CD8^+^ T cells in HNSCC patients is generally associated with a good prognosis (Fridman et al., 2017). Moreover, PD-1+ CD8^+^ T cells showed excellent anti-tumor effect in an anti-PD1-resistant murine HNSCC model (Xu et al., 2020). Therefore, there is an urgent need to explore the molecular mechanisms associated with CD8^+^ T cells infiltration.

Single-cell RNA sequencing (scRNA-seq) has been the subject of rapid technological developments in the last decade, thereby resulting in significant improvements in describing and defining the tumor heterogeneity at a single-cell level (Qi et al., 2019). Besides, application of scRNA-seq to characterize the tumor microenvironment (TME) may provide valuable insights into immune landscapes and even effective immunotherapy strategies (Kurten et al., 2021). Similarly, the gene signature identified based on immune molecular characteristics might be a strong predictor of clinical outcome and immunotherapy response (Song et al., 2022). However, the predictive potential of the molecular mechanisms describing immunophenotypic features in HNSCC have not yet been elucidated.

This study explored the mechanism associated with infiltration of CD8^+^ T cells through integrating bulk and scRNA sequencing. Specifically, a LASSO-Cox regression risk model was built and verified based on the hub immune-related genes (IRGs) identified by weighted gene co-expression network analysis (WGCNA) (Langfelder and Horvath, 2008). Next, we comprehensively represented the various immune features of an 8-gene signature using “ESTIMATE” (Yoshihara et al., 2013), “CIBERSORT” (Newman et al., 2015), single sample gene set enrichment analysis (ssGSEA) approaches, and immunophenoscore (IPS) data. It is expected that the identified risk score will not only be used as an efficient indicator for HNSCC prognosis, but also as a potential therapeutic target.

## Materials and methods

**FIGURE 1:**
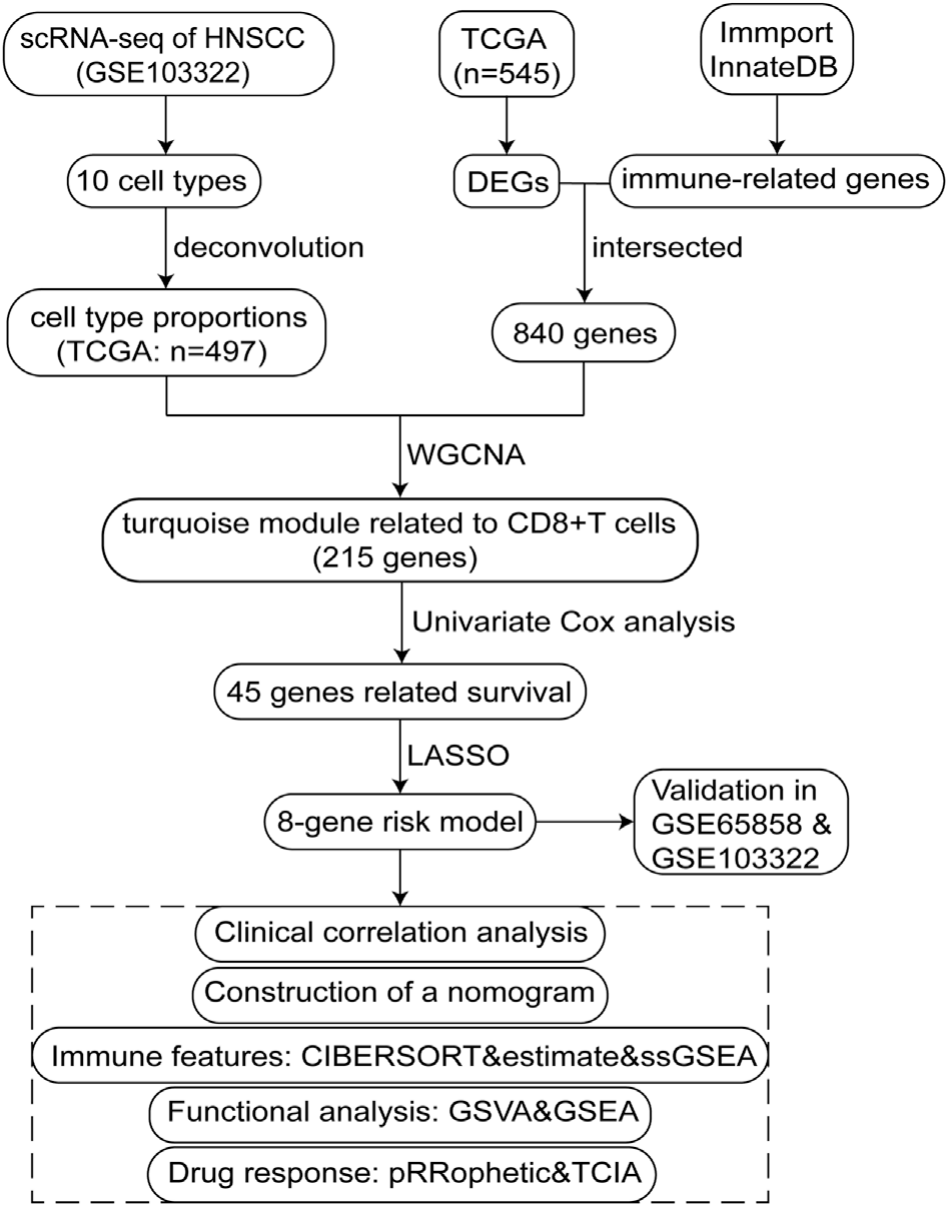
Flow chart schematic of this study.

The study design is illustrated using a flow diagram (Figure 1).

### Data acquisition

The single cell RNA-sequencing profile of GSE103322 dataset (Puram et al., 2017), comprising 5,902 single cells of 18 patients, was downloaded from the Gene Expression Omnibus (GEO) database (https://www.ncbi.nlm.nih.gov/geo/) (accessed date 13 October 2021). HNSCC RNA-sequencing, clinical and mutation data were downloaded from The Cancer Genome Atlas (TCGA) database using the GDC Data Portal (https://portal.gdc.cancer.gov/ (accessed date 13 October 2021). The Fragments per Kilobase per Million (FPKM) values were first converted to transcripts per million kilobase (TPM) values. To validate the prognostic power of the model, the transcriptome and clinical files of the GSE65858 dataset, containing 270 HNSCC samples, were obtained from the GEO database (Wichmann et al., 2015). Notably, a total of 2,720 IRGs were obtained from the ImmPort (https://www.immport.org/home) and InnateDB (https://www.innatedb.com/) databases (accessed date 13 October 2021).

### Processing of single-cell and bulk RNA-seq files

The “Seurat” (version 4.1.1) package in R (version 4.1.2) was applied to group 5,902 cells into appropriate clusters, with the resolution set to 0.8. Results were presented by employing the T-distributed stochastic neighbor embedding (t-SNE) for dimension reduction. Next, diverse cell types, B/plasma cells, endothelial cells, regulatory T cells (Treg cells), mast cells, CD8^+^ T cells, epithelial cells, dendritic cells, macrophages, fibroblasts, and CD4^+^ T cells were identified based on their specific markers. The “Cellchat” (version 1.1.3) package was used to analyze the cell–cell communication, and then deconvolution was performed using the “BisqueRNA” (version 1.0.5) method (Jew et al., 2020) to calculate the cells fractions of TCGA bulk profiles. Based on the TCGA RNA-seq profiles, differentially expressed genes (DEGs) were identified with FDR < 0.05 and |log_2_FC| > 1 set as the cutoff values.

### Determination of immune-related candidate genes

The differential IRGs were determined by overlapping DEGs and IRGs, and then used to screen the hub genes by WGCNA (version 1.7.0). First, Pearson correlation coefficient was determined for every gene, and a suitable soft threshold *β* was automatically selected through the pick Soft Threshold function. Next, gene expression similarity matrix was transformed into an adjacency matrix using a network type of signed and soft powers *β* = 3, followed by employing TOM (topological overlap measure) to cluster genes into network modules. The 1-TOM (dissimilarity TOM) was then applied as the input for hierarchical clustering and the “DynamicTreeCut” algorithm was employed to detect modules (clusters of highly interconnected genes) as branches of the dendrogram. Finally, we identified and selected a module (215 genes) that significantly correlated with CD8^+^ T cells content. Kaplan–Meier (KM) survival and univariate Cox analysis were utilized to determine the hub genes associated with survival at a threshold of *p* < 0.05.

### Development of a prognostic signature in TCGA (*n* = 498)

LASSO-Cox analysis was performed using “glmnet” package to determine the optimal prognostic gene set. The risk score of each HNSCC patient was determined as the sum of normalized gene expression values weighted by their LASSO-Cox coefficients in accordance with the following formula:

$$\mathrm{risk score}=\sum_{i=1}^{n}Coef_{i}^{\text{∗}}Exp_{i}$$

Where $Coef_{i}$ indicates the calculated regression coefficient of each gene in the LASSO-Cox model and $\;Exp_{i}$ represents the mRNA expression value. Kaplan-Meier (KM) analysis, receiver operating characteristic (ROC) curves, and univariate and multivariate Cox regression analyses were employed to validate the independent prognostic factors in TCGA-HNSC and GSE65858 datasets. For better clinical prediction of HNSCC patient survival probabilities, a nomogram was constructed using the “rms” R package based on multivariate Cox analysis results. The concordance index (C-index) of the nomogram was calculated to assess the discriminative ability.

### Immune features and therapy prediction in distinct risk groups

“CIBERSORT” (version 1.03) and “ESTIMATE” (version 1.0.13) analyses were applied to determine the abundance of 22 immune cells and immune infiltration scores. The ssGSEA approach was employed via the “GSVA” (version 1.42.0) package to compute the enrichment scores of 29 immune features (Hänzelmann et al., 2013). To predict the susceptibility of eight common chemotherapeutic drugs (5-Fluorouracil, bleomycin, cetuximab, cisplatin, docetaxel, methotrexate, rapamycin, and sunitinib) for HNSCC, the “pRRophetic” (version 0.5) method was performed to evaluate the half-maximal inhibitory concentration (IC50) of patients in distinct groups (Geeleher et al., 2014). The immunophenoscore (IPS) of HNSCC patients, which is a scoring scheme that characterizes the determinants of tumor immunogenicity (Charoentong et al., 2017), were downloaded from The Cancer Imaging Archive (TCIA) database (https://tcia.at/home, accessed date 15 November 2021). To predict the anti-CTLA4 and anti-PD1 responses, patients with different IPS were further compared between the two risk groups. Finally, the “Maftools” (version 2.10.05) (Mayakonda et al., 2018) package was used to determine the tumor mutational burden (TMB) and identify the driver genes.

### Enrichment analysis

The reference gene sets of Kyoto Encyclopedia of Genes and Genomes (KEGG) pathway (c2. cp.kegg.v7.5.1. symbols.gmt) were obtained from the MSigDB database (https://www.gsea-msigdb.org/gsea/msigdb, accessed date 15 November 2021). GSEA software (version 4.2.3) and Gene Set Variation Analysis (GSVA) were conducted to determine the KEGG pathways with FDR < 0.05.

## Results

### Cell typing in head and neck squamous cell carcinoma scRNA-seq and deconvolution in the Cancer Genome Atlas-HNSC

**FIGURE 2:**
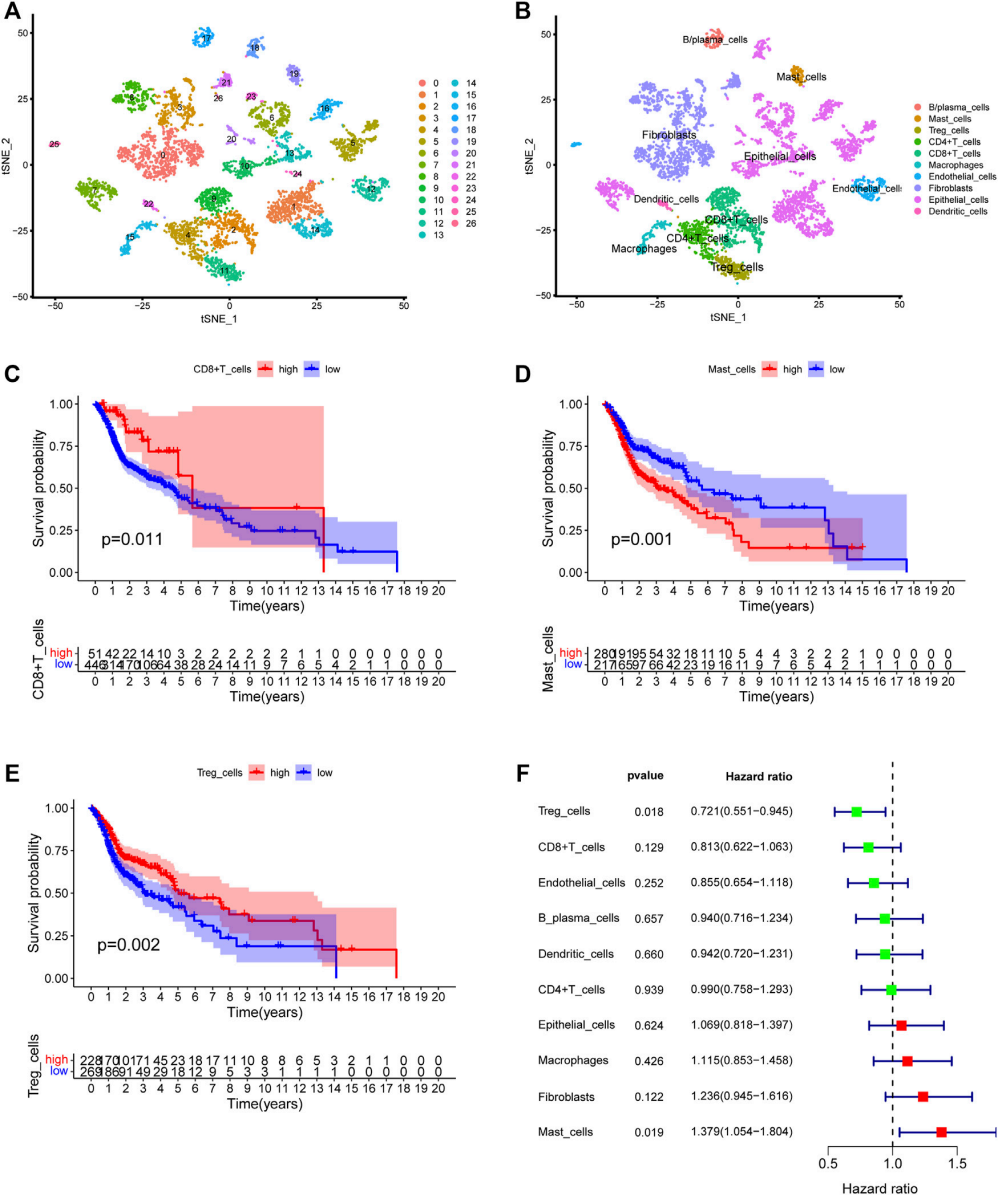
Identification of the HNSCC-associated cell subtypes. **(A)** t-SNE plot classified cell clusters based on scRNA sequencing data. **(B)** t-SNE plot identified the various cell subtypes. **(C–E)** Kaplan-Meier survival analysis of three cell subtypes using the deconvolved TCGA data. **(C)** CD8^+^ T cells: *p* = 0.011, **(D)** Mast cells: *p* = 0.001, **(E)** Treg cells: *p* = 0.002. **(F)** Univariate analysis of ten cell subtypes.

**FIGURE 3:**
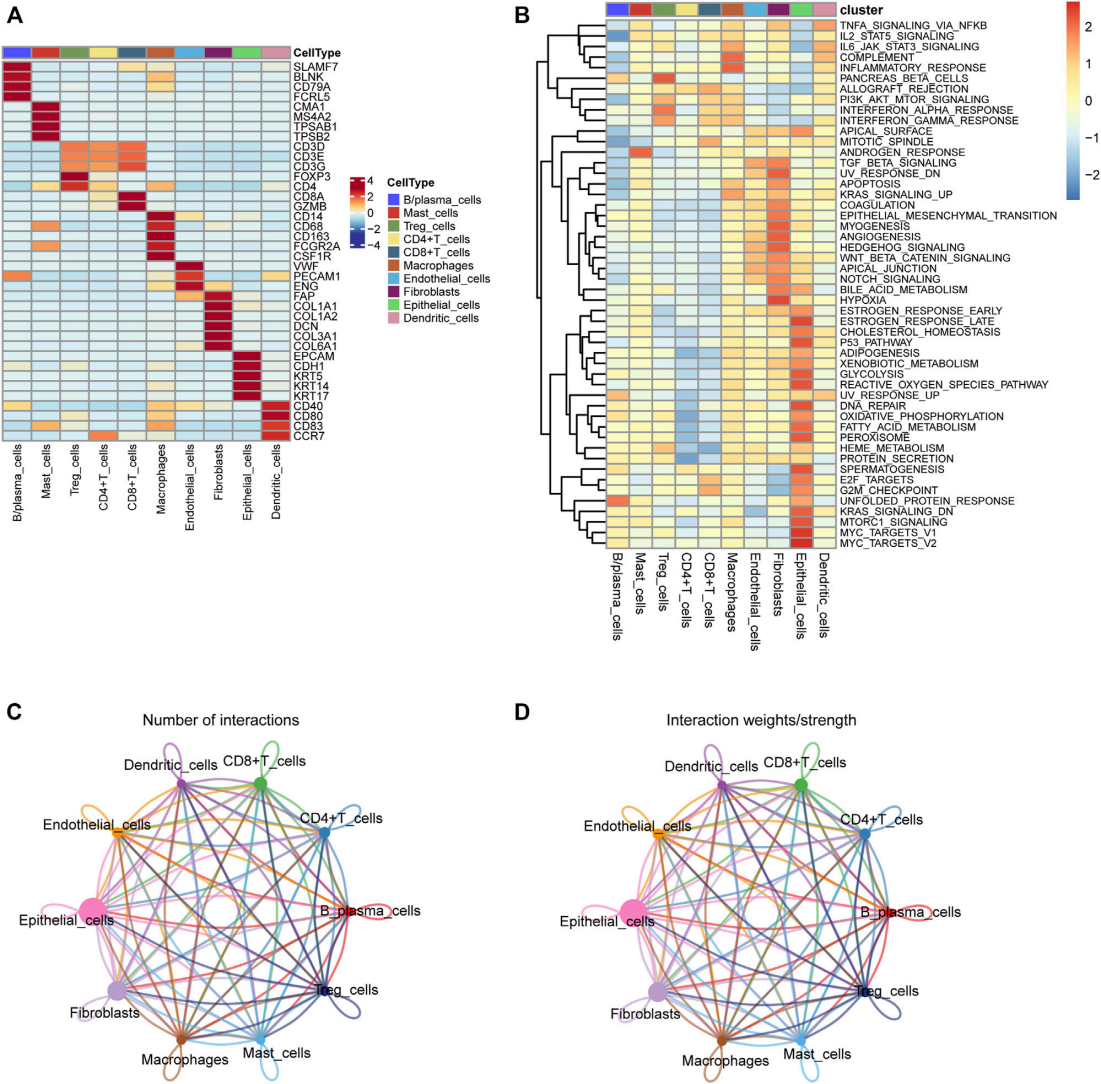
**(A)** The heatmap depicting marker genes associated with ten cell subtypes. **(B)** GSVA enrichment analysis of the cell subtypes. **(C,D)** Cell-cell communication network of ten cell subtypes.

We first collected the Smart-seq2 profile data of 5,902 cells in the GSE103322 dataset. Principal component analysis (PCA) and t-SNE analysis identified 27 cell clusters (Figure 2A). According to expressions of marker genes, 10 distinct cell clusters were identified, including CD8^+^ T cells, macrophages, CD4^+^ T cells, fibroblasts, endothelial cells, B/plasma cells, mast cells, Treg cells, epithelial cells, and dendritic cells (Figures 2B, 3A). GSVA results showed that “MYC_TARGETS_V2” and “MYC_TARGETS_V1” were activated in epithelial cells, whereas “HYPOXIA” was abundant in fibroblasts (Figure 3B). Results obtained after applying the “CellChat” method showed that there was a strong connectivity between different cell types (Figures 3C,D). Next, the BisqueRNA approach was performed to calculate proportions of the 10 cell types by deconvoluting the TCGA bulk profiles. Supplementary Table S1 shows proportion of the 10 cell types in 497 samples. Survival analysis demonstrated that mast cells (*p* = 0.001), CD8^+^ T cells (*p* = 0.011), and Treg cells (*p* = 0.002) were significantly associated with HNSCC outcome (Figures 2C–E). Moreover, univariate Cox analysis indicated that Treg cells were associated with good outcome (*p* = 0.018), whereas mast cells were intimately linked to poor prognosis (*p* = 0.019) (Figure 2F).

### Construction and validation of a gene risk signature associated with CD8^+^ T cells

**FIGURE 4:**
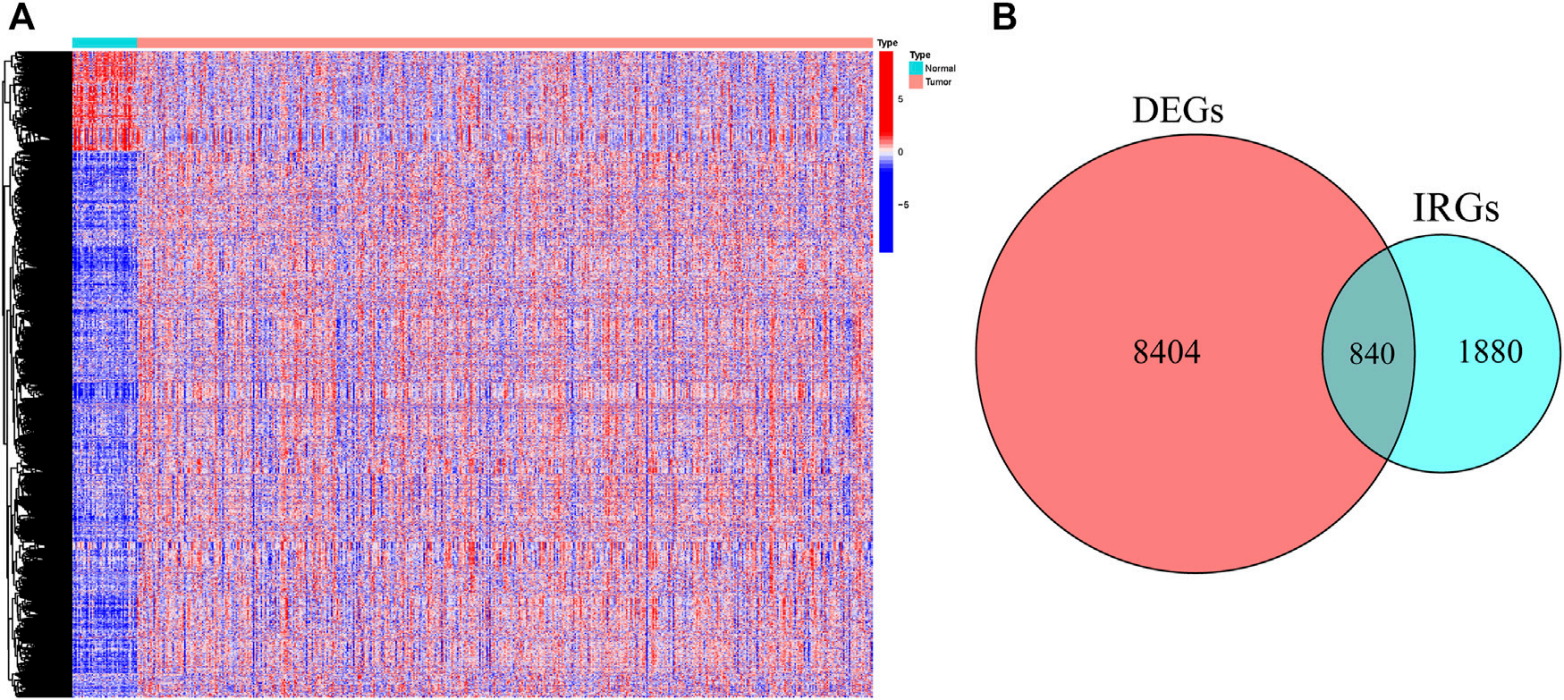
The heatmap **(A)** and Venn diagram **(B)** identified the differentially expressed genes (DEGs) and immune-related DEGs between tumor and normal samples in TCGA.

**FIGURE 5:**
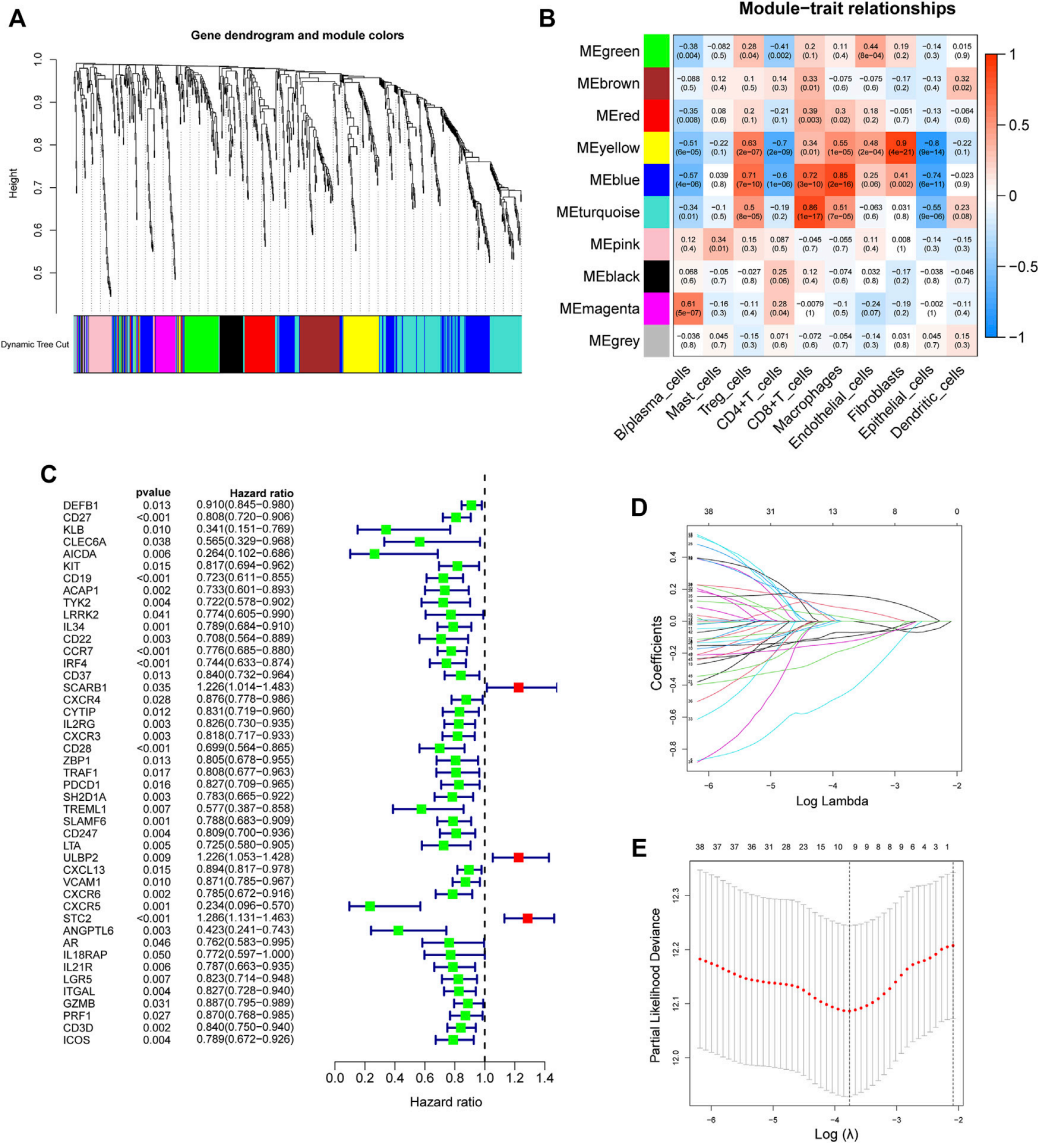
Development of an 8-gene signature. **(A)** The Cluster dendrogram of co-expression network modules obtained by WGCNA. **(B)** Correlation heatmap among ten co-expression modules and the levels of cell subtypes. The turquoise module had the greatest correlation with CD8^+^ T cells (r = 0.86, p = 1e-17). **(C)** Univariate analysis of 45 immune-related hub genes. **(D)** LASSO coefficient profiles of 45 immune-related genes. **(E)** Tuning parameter selection in the LASSO model using ten-time cross-validation.

**FIGURE 6:**
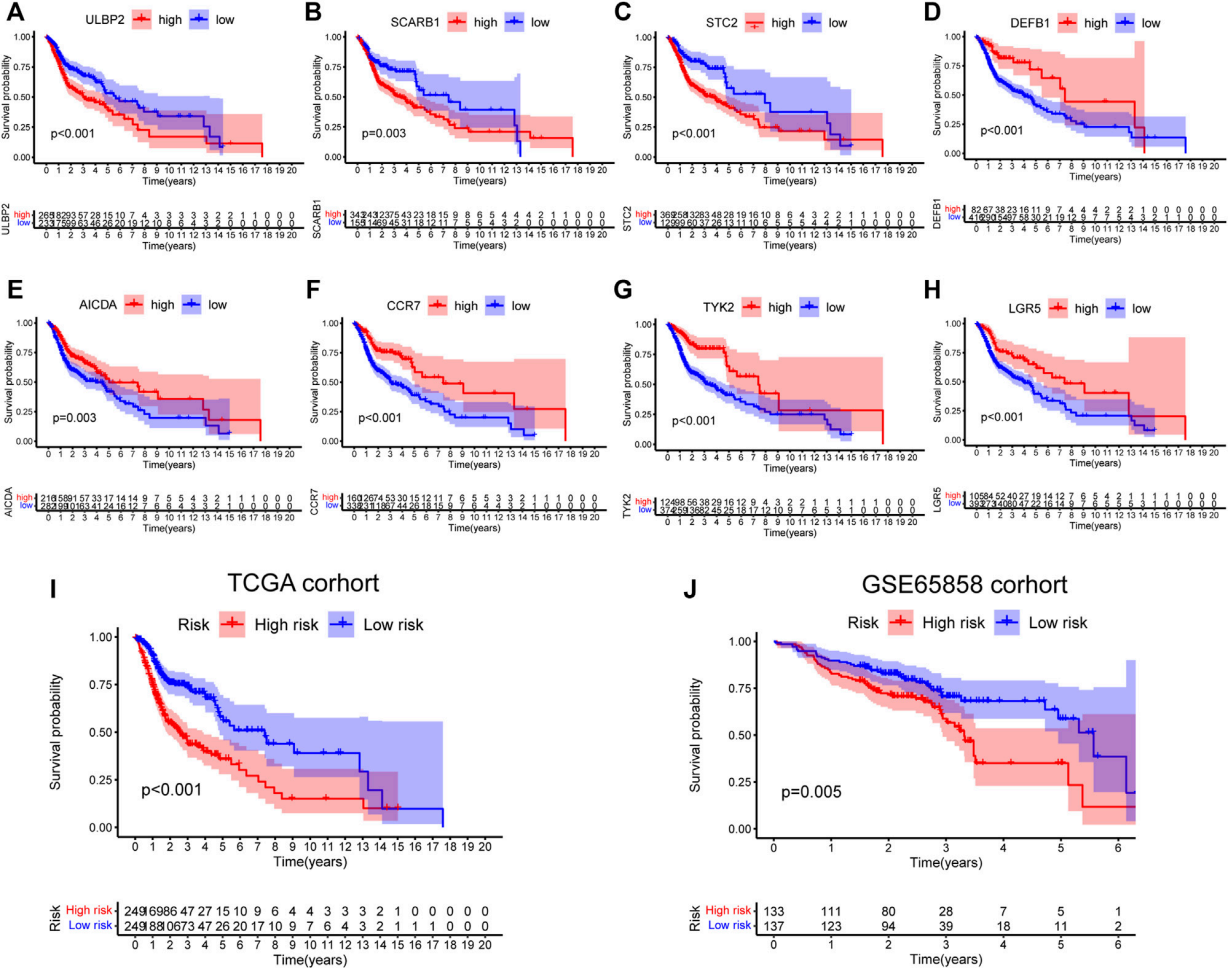
Survival analysis of eight genes in risk signature. **(A)** ULBP2: *p* < 0.001. **(B)** SCARB1: *p* = 0.003. **(C)** STC2: *p* < 0.001. **(D)** DEFB1: *p* < 0.001. **(E)** AICDA: *p* = 0.003. **(F)** CCR7: *p* < 0.001. **(G)** TYK2: *p* < 0.001. **(H)** LGR5: *p* < 0.001. **(I,J)** Survival analysis of the 8-gene signature in TCGA (*p* < 0.001) and GSE65858 cohorts (*p* = 0.005).

First, 9,244 DEGs were obtained from the TCGA-HNSC dataset comprising 501 tumor and 44 normal samples (Figure 4A). Subsequently, 2,720 IRGs from ImmPort and InnateDB databases were matched with DEGs, from which 840 differentially expressed IRGs were obtained for further analysis (Figure 4B). Based on the 840 IRGs and proportions of the 10 cell types in TCGA, the weighted gene co-expression network was generated using the soft-thresholding power *β* = 3, which resulted in identification of 10 modules (Figures 5A,B). To further explore the features of CD8^+^ T cells infiltration, we selected the turquoise module (215 genes) which had the strongest correlation with CD8^+^ T cells (r = 0.86, p = 1e-17). Univariate Cox analysis demonstrated that 45 of the 215 hub genes were closely associated with HNSCC survival (Figure 5C). Therefore, the 45 genes were subjected to LASSO regression analysis to identify the optimal penalty coefficient (Figures 5D,E). The survival analysis identified eight genes, including *DEFB1*, *AICDA*, *TYK2*, *CCR7*, *SCARB1*, *ULBP2*, *STC2*, and *LGR5*, which were significantly associated with HNSCC prognosis (Figures 6A–H). The eight risk regression coefficients were then employed to compute individual risk score of HNSCC patients according to the following formula:

$$\text{Risk score}={\left(-0.097\right)}^{\text{∗}}\text{DEFB}1+{\left(-0.444\right)}^{\text{∗}}\text{AICDA}+{\left(-0.175\right)}^{\text{∗}}\text{TYK}2+{\left(-0.071\right)}^{\text{∗}}\text{CCR}7+0.020^{\text{∗}}\text{SCARB}1+0.079^{\text{∗}}\text{ULBP}2+0.161^{\text{∗}}\text{STC}2+{\left(-0.128\right)}^{\text{∗}}\text{LGR}5$$

Next, the 498 HNSCC patients were stratified into high- and low-risk groups based on the median risk score. KM survival analysis results indicated that the high-risk group patients showed poorer outcomes compared to the low-risk group (*p* < 0.001, Figure 6I). Consistently, similar results were observed in the GSE65858 dataset (*p* = 0.005, Figure 6J).

### Validation in the Cancer Genome Atlas-HNSC and GSE65858 cohorts, and scRNA-seq data

**FIGURE 7:**
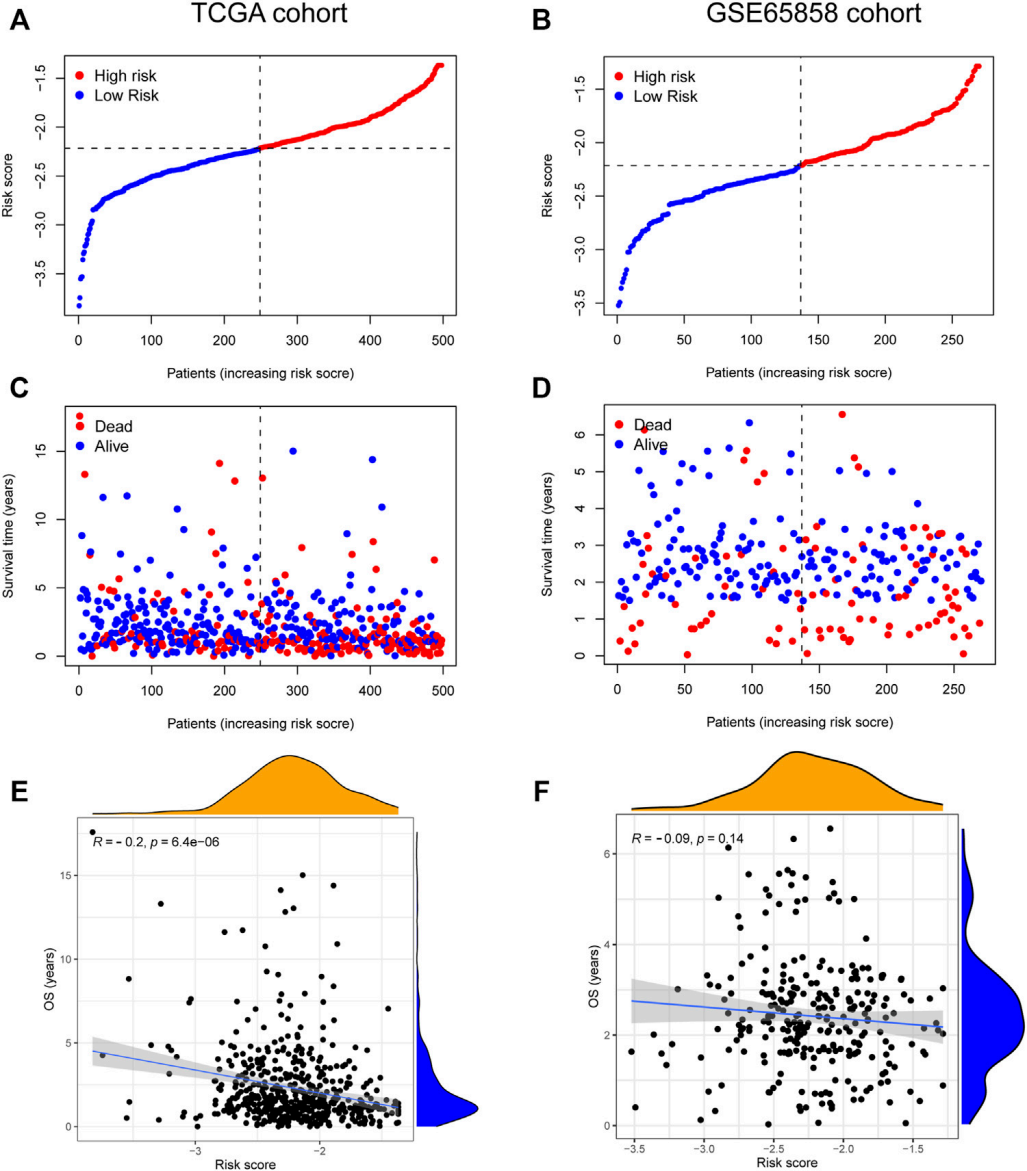
The relationship between risk score and HNSCC survival. **(A–D)** Distribution of risk score and survival status of 8-gene signature in TCGA **(A,C)** and GSE65858 **(B,D)** cohorts. **(E,F)** The correlation analysis between overall survival (OS) and risk score in TCGA **(E)** and GSE65858 **(F)** cohorts.

**FIGURE 8:**
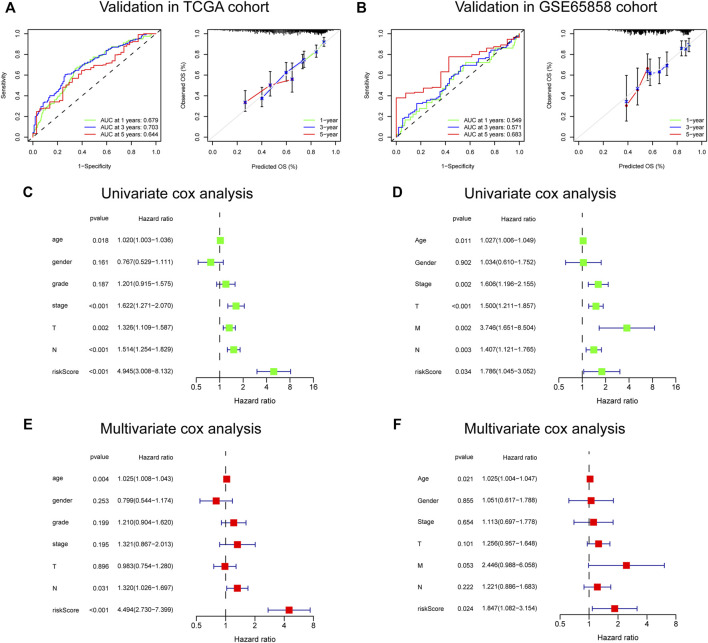
Validation of the 8-gene signature in TCGA and GSE65858 cohorts. **(A,B)** The ROC and calibration curves for determining the accuracy of model. **(C–F)** Univariate and multivariate analysis of clinical features and risk score.

**FIGURE 9:**
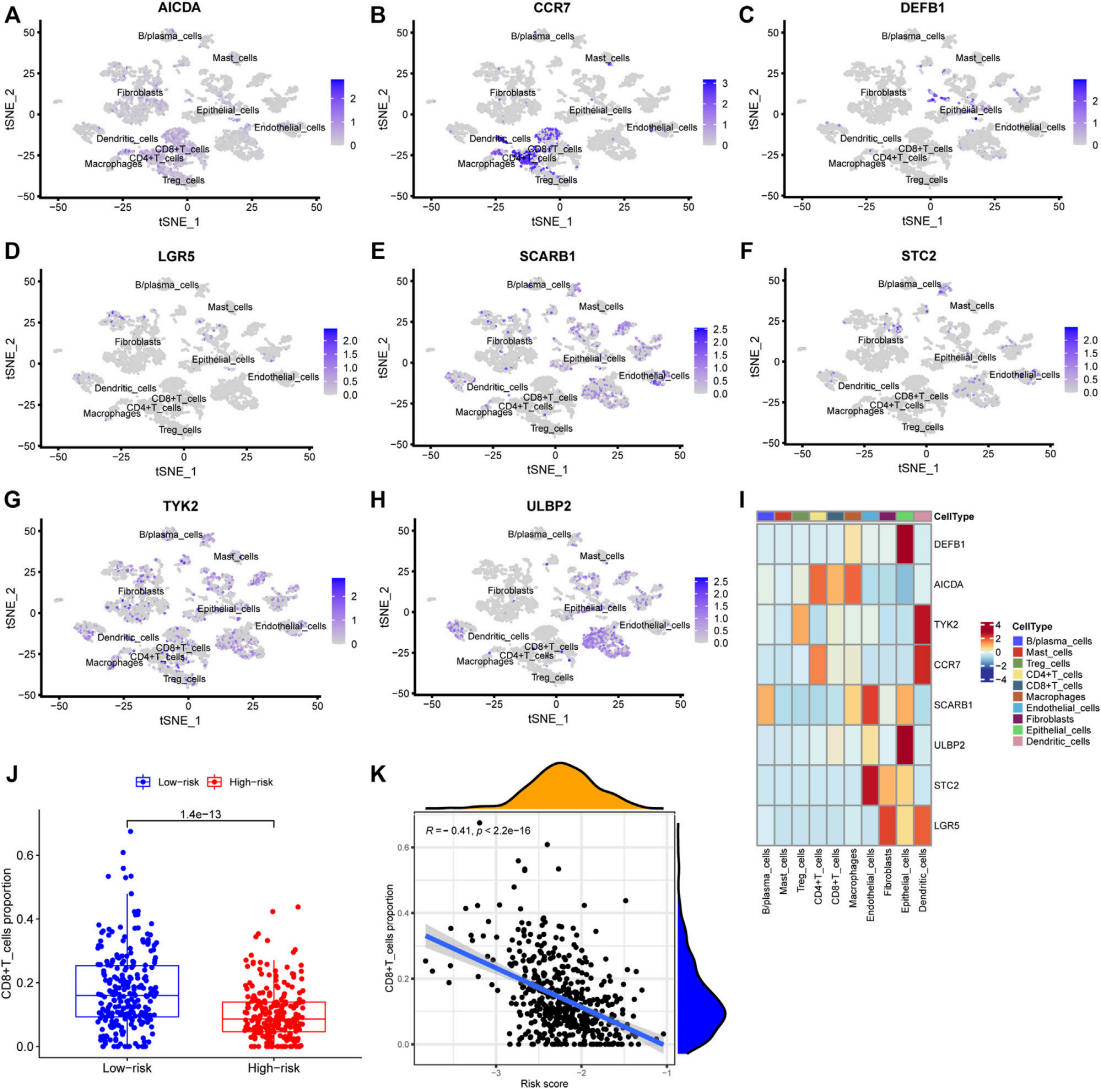
Verification using single-cell sequencing data. **(A–H)** Colors indicating the localization of the expression of eight genes: AICDA, CCR7, DEFB1, LGR5, SCARB1, STC2, TYK2, and ULBP2. **(I)** Heatmap depicting expressions of the eight genes in the cell subtypes. **(J)** The levels of CD8^+^ cells in TCGA deconvoluted data between low- and high-risk groups (*p* = 1.4 e-13). **(K)** Correlation analysis between CD8^+^ T cells levels and risk score (r = −0.41, *p* < 2.2 e-16).

The risk score, survival status distributions of HNSCC patients, and correlation analysis are displayed in Figure 7. Results demonstrated that survival reduced with rising risk score, and there was a significant correlation between risk score and survival in TCGA cohort (r = -0.2, *p* = 6.4e-06). Time-dependent ROC and calibration curves at one-, three-, and five-years were then constructed (Figures 8A,B). In the TCGA cohort, the areas under the ROC curves (AUCs) were 0.679, 0.703, and 0.644 for 1-, 3-, and 5-years survival, respectively. In both the TCGA and GSE65858 cohorts, univariate and multivariate Cox analyses demonstrated that the risk score was an independent predictor for prognosis (Figures 8C–F). To determine the cells that these eight genes were enriched, the distribution plots for expressions of the eight genes in the 10 cell types identified in the GSE103322 dataset were generated and are shown in Figures 9A–I. Results showed that the expression levels of *DEFB1* and *ULBP2* were higher in epithelial cells, whereas *TYK2* and *CCR7* levels were abundant in dendritic cells. In addition, the endothelial cells had higher expressions of *SCARB1* and *STC2*, and *LGR5* was highly expressed in both dendritic cells and fibroblasts. Based on proportions of the 10 cell types obtained after deconvolution, correlation analysis was performed to evaluate the association among proportion of CD8^+^ T cells and risk score. Obtained results revealed that fractions of CD8^+^ T cells declined as the risk score increased (r = −0.41, *p* < 2.2 e-16, Figures 9J,K).

### Construction of a nomogram for clinical practice

**FIGURE 10:**
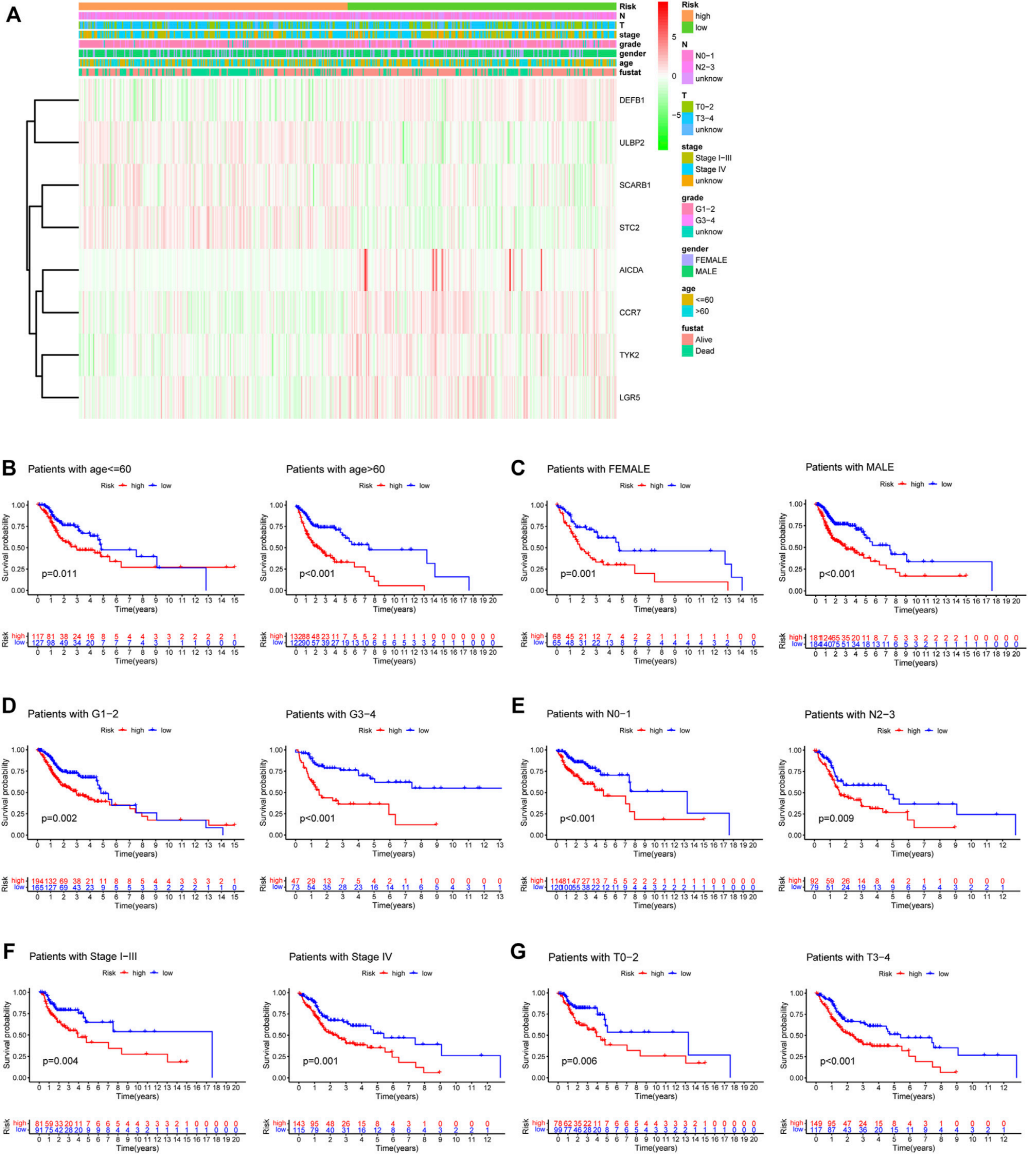
The relationship between risk signature and the clinical characteristics. **(A)** The heatmap depicting eight gene expressions among distinct clinical patterns. **(B–G)** Kaplan-Meier survival analysis according to the 8-gene signature stratified by clinicopathological factors. **(B)** age<=60: *p* = 0.011, age>60: *p* < 0.001. **(C)** Female: *p* = 0.001, Male: *p* < 0.001. **(D)** G1-2: *p* = 0.002, G3-4: *p* < 0.001. **(E)** N0-1: *p* < 0.001, N2-3: *p* = 0.009. **(F)** Stage I-III: *p* = 0.004, Stage IV: *p* = 0.001. **(G)** T0-2: *p* = 0.006, T3-4: *p* < 0.001.

**FIGURE 11:**
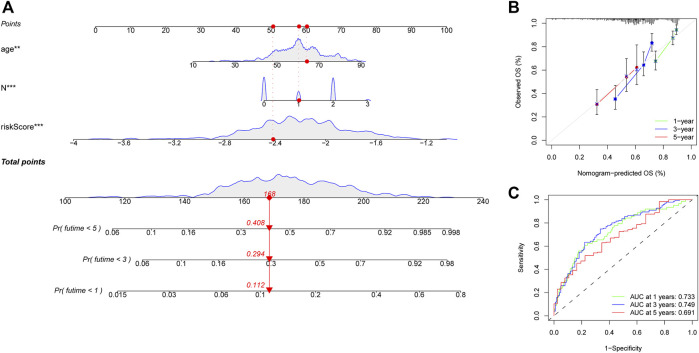
Construction of a nomogram for predicting survival of HNSCC patients. **(A)** Nomogram using two clinical traits (N stage and age) and the risk score. **(B,C)** The calibration and ROC curves for determining the reliability of the nomogram to predict one-, three-, and five-year survival rates.

A heatmap was generated to depict the changes in expression of the eight genes between different clinical subgroups (Figure 10A). The performance of the risk score was then explored in different clinicopathological subgroups, including clinical stage (stage I-III and stage IV), age (<=60 and >60), grade (G1-2 and G3-4), T stage (T0-2 and T3-4), N stage (N0-1 and N2-3), and gender (female and male). According to the survival analysis results, HNSCC patients with high-risk scores consistently had a poorer outcome in all subgroups (Figures 10B–G). Next, the three remarkable variables in the multivariate analysis, including age, N stage, and risk score, were selected and used to build a nomogram (C-index: 0.676) for estimating the 1-, 3-, and 5-year survival rate (Figure 11A). By drawing a vertical line to the axis points, we could estimate patient survival based on total points. Overall, the calibration curves and the AUC’s (1-, 3-, and 5-year: 0.733, 0.749, and 0.691, respectively) suggested that the risk model could accurately predict the HNSCC survival rate (Figures 11B,C).

### The immune landscape of the two risk groups

**FIGURE 12:**
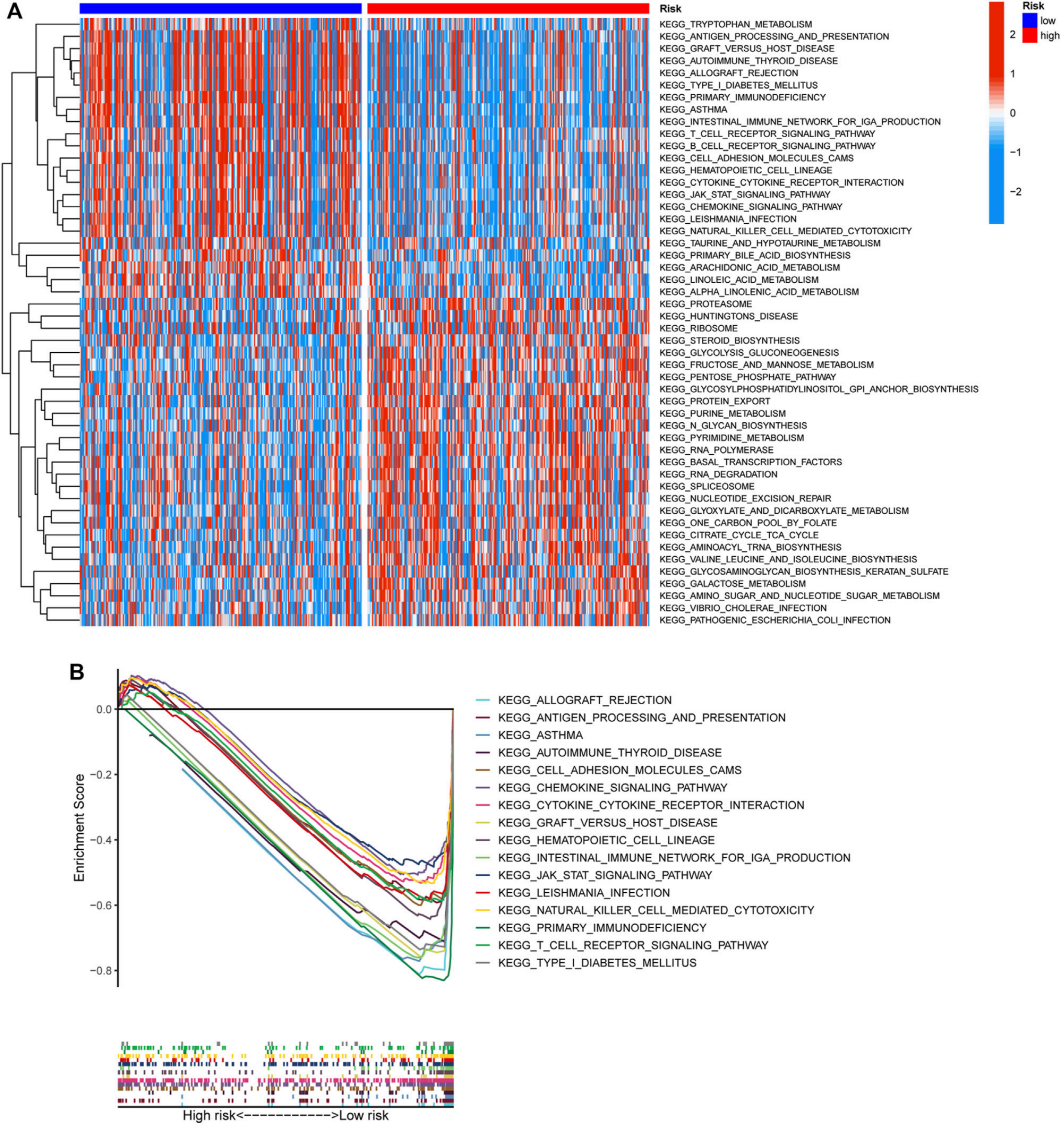
Functional enrichment characteristics of the risk signature. **(A)** Different activities of KEGG pathway scored by GSVA between high- and low-risk groups. **(B)** GSEA analysis showing the sixteen KEGG functional pathways enriched in low-risk group.

**FIGURE 13:**
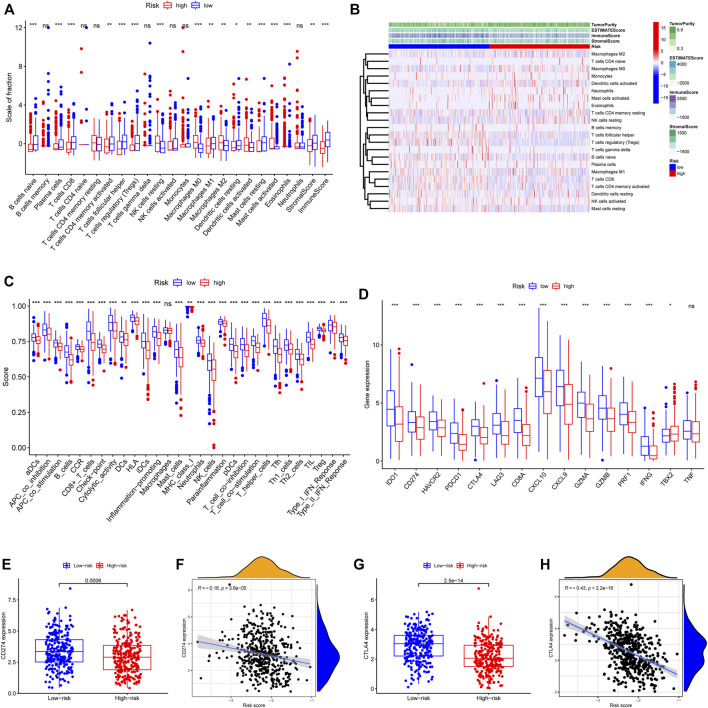
Patterns of immune cells infiltration in two risk groups. **(A)** The box plot showing the fractions of 22 infiltrating immune cells and immune-related scores based on CIBERSORT and ESTIMATE algorithms. **(B)** A heatmap presenting the 22 immune cells in the two risk score subgroups with different immune-related scores. **(C)** The ssGSEA scores for 29 immune gene sets. **(D)** Differential expressions of the 15 immune checkpoint-related genes. **(E)** CD274 expression difference among the high- and low-risk groups (*p* = 0.0006). **(F)** The spearman correlation plot between CD274 expression and risk score (r = −0.18, *p* = 3.6 e-05). **(G)** CTLA4 expression difference among the high- and low-risk groups (*p* = 2.5 e-14). **(H)** The spearman correlation plot between CTLA4 expression and risk score (r = −0.43, *p* < 2.2 e-16). ∗*p* < 0.05; ∗∗*p* < 0.01; ∗∗∗*p* < 0.001; ns: no significance.

**FIGURE 14:**
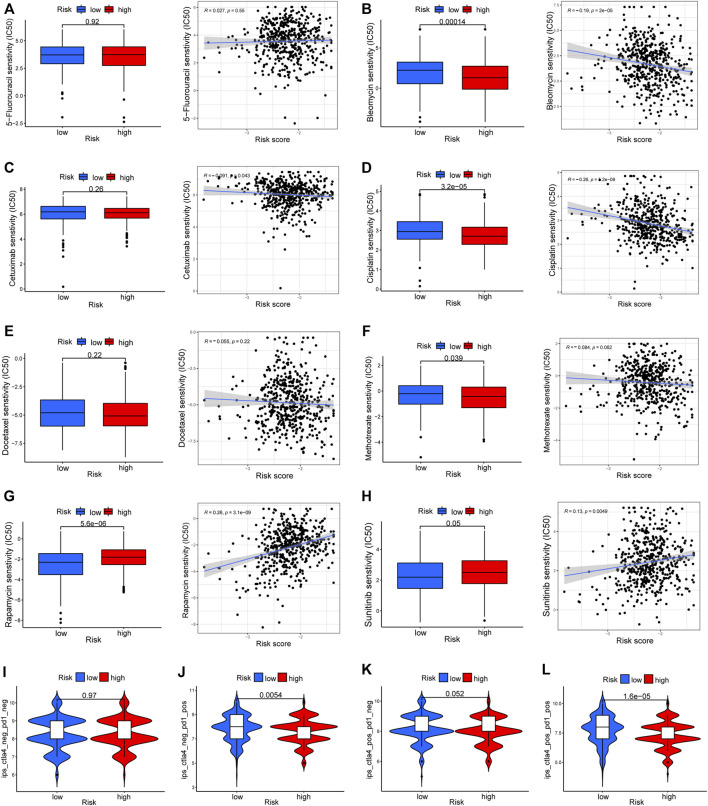
Drug response prediction between the two risk groups. **(A–H)** The IC50 of eight common chemotherapeutic agents (5-Fluorouracil, Bleomycin, Cetuximab, Cisplatin, Docetaxel, Methotrexate, Rapamycin, and Sunitinib) and correlation analysis with risk score. **(I–L)** The difference of immunophenoscore (IPS) scores among high- and low-risk groups.

**FIGURE 15:**
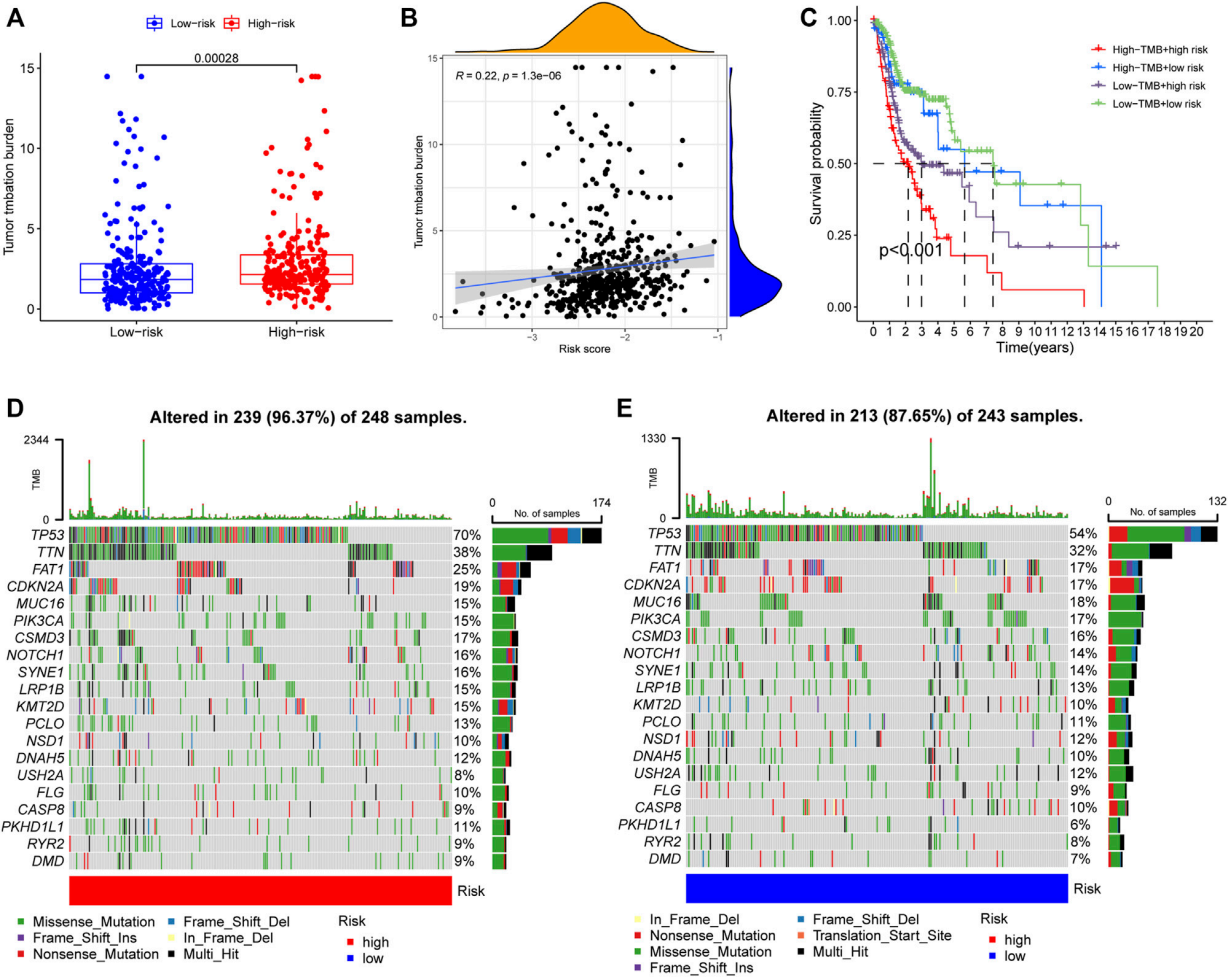
**(A)** TMB difference among the high and low risk groups. *p* = 0.00028. **(B)** The Spearman correlation analysis between risk score and TMB. r = 0.22, *p* = 1.3e-06. **(C)** K-M survival analysis stratified by both TMB and risk scores. *p* < 0.001. **(D,E)** Distribution of the top 20 variant mutated genes among high **(D)** and low **(E)** risk groups. The waterfall plot showing the genetic alterations types.

To elucidate the biological characteristics activated in distinct risk groups, KEGG pathway enrichment analysis was performed using GSVA and GSEA methods. By setting the adjusted *p* value (FDR) < 0.05, a total of 51 and 16 pathways were obtained in GSVA and GSEA, respectively (Figures 12A,B). Several overlapping immunoregulatory processes were enhanced in the low-risk group, including “hematopoietic cell lineage”, “T cell receptor signaling pathway”, “antigen processing and presentation” and “natural killer cell-mediated cytotoxicity”. To describe the patterns of immune infiltrations, CIBERSORT and ESTIMATE methods were implemented for calculating the cell fractions and immune-related scores of HNSCC samples (Figures 13A,B). The low-risk group showed more significant infiltrations of CD8^+^ T cells, M1 macrophages, follicular helper T cells, plasma cells, regulatory T cells, and memory activated CD4^+^ T cells, as well as a higher immune score. With regard to the high-risk group, abundant infiltrations of activated mast cells, M2 macrophages, resting NK cells, and low immune score were observed. The ssGSEA approach was then applied to estimate the scores of specific immune functions and cells. Results revealed significant differences of most immune cells and functions between high- and low-risk groups (Figure 13C). Besides, 15 immune checkpoint molecules (IFNG, GZMB, HAVCR2, CD274, CD8A, PDCD1, TBX2, IDO1, GZMA, LAG3, CXCL10, CTLA4, PRF1, CXCL9, and TNF) were selected and their expressions were compared between the two risk groups (Figure 13D). Based on the correlation analysis results, it was evident that the expressions of CD274 and CTLA4 in the two groups were significantly different (CD274: *p* = 0.0006; CTLA4: *p* = 2.5e-14), and decreased as the risk score rose (CD274: r = −0.18, *p* = 3.6 e-05; CTLA4: r = −0.43, *p* < 2.2 e-16) (Figures 13E–H). Next, the pRRophetic algorithm was applied to predict the IC50 of eight common chemotherapeutic drugs between the two groups. Patients with a high-risk score showed an increased susceptibility to bleomycin (*p* = 0.00014), cisplatin (*p* = 3.2e-05), and methotrexate (*p* = 0.039). On the other hand, low-risk group patients showed increased sensitivity to rapamycin (*p* = 5.6e-06) (Figures 14A–H). To forecast the response to anti-PD1 and anti-CTLA4 immunotherapy, the IPS scores of HNSCC patients were used to compare the two risk groups (Figure 14I–L). Results indicated that patients in the low-risk group exhibited higher IPS scores and showed greater response to anti-PD1 therapy and anti-PD1 plus anti-CTLA4 therapy (ips_ctla4_neg_pd1_pos: *p* = 0.0054, ips_ctla4_pos_pd1_pos: *p* = 1.6e -05) relative to patients in the high-risk group. Given the important role of TMB in prognosis, the intrinsic connection between TMB and risk score was explored to assess genetic signature. It was found that the high-risk group exhibited higher TMB (Figure 15A). A significant correlation was observed between TMB and risk score (r = 0.22, *p* = 1.3e-06, Figure 15B). Survival curve suggested that a low TMB/low risk group showed a great outcome compared with the other groups (*p* < 0.001, Figure 15C). The top 20 driver genes with the highest alteration frequency were analyzed (Figures 15D,E) and four genes (*TP53*, *PKHD1L1*, *DNAH9*, *FAT1*) were significantly different between high- and low-risk groups (Supplementary Table S2).

## Discussion

Immunotherapy has been successful used to treat cancer patients in the advanced tumor stage. Nevertheless, clinical application of the strategy is hampered by several limitations, including low response rates, development of serious side effects, and drug resistance (Sacco et al., 2021). One of the key reasons for these limitations is the paucity of potential predictive markers. In the present study, we calculated the proportion of CD8^+^ T cells, and selected IRGs-related to CD8^+^ T cells infiltration by integrating scRNA and bulk sequencing profiles. As a result, 215 differential IRGs were identified by WGCNA, of which 45 genes were significantly associated with HNSCC survival. Subsequently, we developed and validated an 8-gene risk model which may be useful for predicting prognosis and immunotherapeutic effect.

The eight critical genes, including *DEFB1*, *AICDA*, *TYK2*, *CCR7*, *SCARB1*, *ULBP2*, *STC2*, and *LGR5*, play essential roles in tumor progression and immune-modulatory effects. For example, DEFB1, the human antimicrobial peptide defensin *β* 1, is considered as a potential tumor suppressor gene and has been shown to mediate PI3K/mTOR signaling, thereby leading to death of tumor cells (Sun et al., 2006; Lee et al., 2015). *DEFB1* was also found to be theoretically useful as a prognostic biomarker for HNSCC (Han et al., 2014). Moreover, *DEFB1* was commonly detected in epithelial cells, which is consistent with our results. UL16-binding protein 2 (*ULBP2*), a ligand of the activating NK cell receptor *NKG2D*, was found to be engaged in target recognition by NK cells (Textor et al., 2011). A previous study confirmed that the soluble *ULBP2* secreted by cancer cells contributed to the immune escape (Waldhauer and Steinle, 2006). Herein, we observed that *ULBP2* was upregulated in epithelial cells. Meanwhile, *ULBP2* has been shown to be a prognosis indicator for several cancers, such as lung cancer and pancreatic cancer (Chang et al., 2011; Yamaguchi et al., 2012). The activation-induced cytidine deaminase (*AICDA*) is an essential enzyme of the adaptive immune system. A recent study found that elevated expression of *AICDA* regulates the function of B cells in regional lymph nodes and significantly improves prognosis of HNSCC patients (Pylaeva et al., 2021). Tyrosine kinase 2 (*TYK2*), a member of the Janus kinase (JAK) family, has emerged as both a promising biomarker and a target for anti-cancer therapies (Borcherding et al., 2021). It has been reported that high expression of *TYK2* is associated with better prognosis of HNSCC (Fang et al., 2021). A recent review concluded that CC motif chemokine receptor (*CCR7*) is correlated with good outcomes of HNSCC patients (Korbecki et al., 2020). However, if located on cancer cells, *CCR7* and its ligands (*CCL19*/*CCL21*) is a vital axis for carcinogenic properties, such as epithelial-mesenchymal transition (EMT) tumor invasion and migration (Chen et al., 2020; Korbecki et al., 2020). Notably, the present study found that *CCR7* was predominantly expressed in dendritic cells. *SCARB1* has been demonstrated to be involved in cholesterol metabolism, thereby facilitating cancer progression (Gutierrez-Pajares et al., 2016). In addition, stanniocalcin-2 (*STC2*) exerted a significant role in a wide variety of signaling pathways in HNSCC apoptosis and autophagy (Li et al., 2020). Studies have revealed that downregulated expression of *STC2* can suppress growth of HNSCC cells (Li et al., 2019; Li et al., 2020). Moreover, the leucine-rich repeat-containing G protein-coupled receptor *LGR5* participated in Wnt signaling and was intimately linked to the severity of HNSCC (Dalley et al., 2015).

Given the important role of immune cell infiltrations in the diagnosis and treatment of diseases, we further explored the immune landscape in different HNSCC groups. Based on the degree of immune cell infiltrations, particularly CD8^+^ T cells, the tumor phenotypes can be defined as two major patterns, “hot” and “cold”, which are associated with good and poor antitumor immune responses, respectively (Galon and Bruni, 2019). This study explored the abundance of immune cells and functions using CIBERSORT, ESTIMATE, and ssGSEA methods. According to the obtained results, the low-risk group exhibited more infiltration of CD8^+^ T cells, memory activated CD4^+^ T cells, and plasma cells, as well as higher immune score, and thus can be categorized as “hot” tumor phenotype. On the other hand, the high-risk group showed greater abundance of activated mast cells, resting NK cells, and M2 macrophages, and lower immune score, suggesting the “cold” tumor phenotype. Furthermore, the immune checkpoint-related genes exhibited relatively high expressions in the low-risk group, including *IFNG*, *PRF1*, *GZMA*, *GZMB*, *CXCL10*, *CXCL9*, *CD8A*, *CD274* (*PD-L1*), *HAVCR2*, *IDO1*, *LAG3*, *CTLA4*, and *PDCD1*. Studies have confirmed that infiltration of M2 macrophages is associated with tumorigenic chronic inflammation with secretion of protumorigenic factors, such as *IL-6*, *VEGF*, and *TGFβ* (Ruffell and Coussens, 2015). Accumulating evidence suggests that preexisting CD8^+^ T cells and *PD-L1* expression are generally correlated with improved efficacy of immunotherapy (Farhood et al., 2019; Gavrielatou et al., 2020). Consistently, our results suggested that patients with low-risk score, as a consequence of higher IPS scores, had more vigorous immune responses to anti-PD1 therapy and anti-PD1 plus anti-CTLA4 therapy. Moreover, patients in the two groups exhibited varying sensitivity to four common chemotherapeutic drugs, including bleomycin, cisplatin, methotrexate, and rapamycin (Cramer et al., 2019). Notably, previous studies have verified the therapeutic safety and effectiveness of chemotherapy in combination with *PD-L1* blockade (Burtness et al., 2019; Cohen et al., 2019). Nevertheless, different sensitivities to 5-Fluorouracil, cetuximab, docetaxel, and sunitinib were not observed in this study. TMB level was considered to be an indicator of immunotherapy response (Rizvi et al., 2015). We then examined the relationship between TMB and the risk score. The alteration frequency of *TP53*, *PKHD1L1*, *DNAH9* and *FAT1* was significantly different between high- and low-risk groups. *TP53* is one of the most frequently mutated genes in HNSCC and *TP53* mutations play a critical role in tumorigenesis and progression (Nathan et al., 2022). Understanding the *DNAH9* and *FAT1* mutations may contribute to cancer surveillance and treatment (Huang et al., 2021; Yang et al., 2022). Investigation of the mutational signatures may allow for an improved selection of immunotherapies in individual patients.

However, this study was limited by the fact that it lacked experimental and clinical pathology studies to validate the function of the eight genes. Therefore, further clinical trials are needed to confirm the predictive potential of the risk signature.

## Conclusion

In conclusion, by comprehensively analyzing the single-cell and bulk RNA sequencing of HNSCC, this study developed and externally validated a novel and robust model based on eight CD8^+^ T cells-related genes. It is expected that the 8-gene signature will facilitate understanding of HNSCC immune characteristics, predict prognosis of HNSCC patients, and guide the clinical use of immunotherapy.

## Data Availability

The datasets presented in this study can be found in online repositories. The names of the repository/repositories and accession number(s) can be found in the article/Supplementary Material.
